# Effects of unexpected underfoot perturbations during turning on measures of mediolateral stability and corresponding recovery strategies

**DOI:** 10.1242/jeb.251471

**Published:** 2026-04-24

**Authors:** Tyler K. Ho, Nicholas Kreter, Cameron Jensen, JunSeop Son, Paula Kramer, Peter C. Fino

**Affiliations:** ^1^ University of Utah, Department of Health & Kinesiology, Salt Lake City, UT 84112, USA; ^2^ Center for Limb Loss and MoBility, VA Puget Sound Health Care System, Seattle, WA 98108, USA; ^3^ University of North Carolina-Greensboro, Department of Kinesiology, Greensboro, NC 27412, USA; ^4^ University of Nebraska Omaha, Department of Biomechanics, Omaha, NE 68182, USA

**Keywords:** Dynamic balance, Locomotion, Margin of stability, Gait asymmetry, Reactive balance

## Abstract

Humans regularly walk across uneven terrain, which demands reactive control strategies to maintain forward progress and stability. While reactive control during walking has been well described during straight gait, it is unclear how reactive control differs during turning gait. Because turning is asymmetrical, perturbations to the inside and outside limbs may elicit different reactive adjustments. This study investigates how unexpected underfoot perturbations alter stability measures during turning and how individuals alter their foot placement to maintain stability after such perturbations. Seven healthy adults completed walking trials around a circular track while wearing mechanized shoes that pseudo-randomly delivered underfoot perturbations to either the inside or outside limb. We calculated mediolateral margin of stability corrected for centripetal acceleration (ML MoS_C_), step width and step length from kinematic data. Linear mixed effects models compared the effects of perturbation type (inversion versus eversion) and perturbed limb (inside versus outside) for each outcome measure. ML MoS_C_ was affected by both perturbation type and perturbed limb, with larger changes observed during eversion and outside limb perturbations. Changes to step width and step time during two recovery steps after each perturbation were primarily influenced by the perturbed limb – outside limb perturbations elicited consistent changes during two recovery steps compared with one altered step after inside limb perturbations. Perturbations to the outside limb during turning disrupted gait longer than perturbations to the inside limb. This difference across perturbed limb may indicate that outside steps are more important than inside ones for maintaining and recovering stability during turning.

## Teaser

**Summary:** Perturbations during turning disrupted stability until the first outside step post-perturbation, suggesting outside steps are more important than inside ones for maintaining and recovering stability during turning.

## INTRODUCTION

Complex walking environments challenge human balance – the ability to avoid a fall – on a daily basis. Walking in such environments, which affect one's instantaneous mechanical stability (i.e. the biomechanical state of the body), demands the use of both anticipatory and reactive control strategies because of expected and unexpected perturbations (Bruijn and van Dieen, 2018; Hof and Duysens, 2013; Zheng et al., 2011), with unanticipated perturbations necessitating reactive control strategies. To compensate for these perturbations and stay upright, reactive control strategies utilize changes to foot placement (Bruijn and van Dieen, 2018; Fettrow et al., 2019; Kreter et al., 2022; Reimann et al., 2017), ankle or hip joint torque (Hof et al., 2010), push-off force in the trailing limb (Jin et al., 2023), or step time (Roeles et al., 2018). Such studies examining reactive balance control during straight-line gait have provided insight into effective locomotor control during walking, but fewer studies have examined reactive balance control during non-straight walking, including curved walking and turning.

As many as half of all steps taken throughout a day are non-straight turning steps (Glaister et al., 2007), and turning differs from straight-line gait in several important ways. During turning, the body center of mass (CoM) is biased toward the medial edge of the base of support (Taylor et al., 2005; Xu et al., 2004). This asymmetry is a hallmark of turning (Orendurff et al., 2006), where steps with the outside limb tend to be longer and narrower compared with those with the inside limb. Because turning gait is asymmetrical, perturbations to the inside and outside limbs may affect the CoM trajectory in different ways (Kreter and Fino, 2024). For example, people may be more likely to experience a slip on the outside stepping limb compared with on the inside stepping limb (Rasmussen et al., 2022). Asymmetrical ground reaction forces and body inclination angles further suggest that the outside limb is more sensitive to irregular walking surfaces than the inside limb during turning (Yamaguchi et al., 2018, 2012).

However, the effects of underfoot perturbations (i.e. uneven walkways or irregular terrain) on turning remain unclear; the majority of uneven walkway tests involve straight walking. Studies that have explored changes in stability due to perturbations during turning tasks have deployed only one perturbation per trial (Kreter and Fino, 2024; Rasmussen et al., 2022), making them more predictable than in everyday life, where perturbations may come unpredictably and in rapid succession. When walking in ecologically valid settings where perturbations are unpredictable and frequent, participants may adopt different anticipatory or reactive strategies to maintain their stability (Liu et al., 2024). Several studies have used shoe-based perturbation devices to achieve ecologically relevant, repeated and unpredictable underfoot ankle inversion and eversion perturbations, and have demonstrated that underfoot inversion and eversion perturbations alter CoM dynamics in different ways (Kim and Ashton-Miller, 2012; Kim et al., 2013; Kreter et al., 2022). However, to date, these studies have similarly been restricted to straight walking. As these underfoot perturbations function by displacing the center of pressure in a medial or lateral direction (Kreter et al., 2022; Nnodim et al., 2013), underfoot inversion or eversion perturbations may disrupt the lateral motion of the CoM, and subsequent stepping responses to control stability, throughout a turn.

The purpose of this study was to explore how unexpected underfoot perturbations affect mediolateral (ML) stability during turning gait. Specifically, we aimed to answer three questions: (1) how do unexpected underfoot perturbations affect metrics of ML gait stability during turning?; (2) does the direction of the perturbation (e.g. inversion versus eversion ankle perturbations) affect the recovery response?; and (3) do perturbations to the inside foot elicit different recovery responses from perturbations to the outside foot? To answer these questions, we measured ML margin of stability (MoS) accounting for centripetal acceleration (ML MoS_C_), step width and step time as healthy young adults walked around a circular platform with unexpected, discrete underfoot perturbations. We hypothesized that unexpected underfoot perturbations would decrease ML MoS_C_ during turning for up to two steps after each perturbation (H1), similar to results seen in straight-line gait (Kreter et al., 2022). We hypothesized that inversion and eversion perturbations would elicit opposite changes in step width and step time on recovery steps following the perturbations (H2). We also hypothesized that perturbations to the outside limb would elicit greater changes to step width and step time than perturbations to the inside limb (H3).

## MATERIALS AND METHODS

### Participants

Seven healthy young adults (3 female/4 male, mean±s.d. age 23.3±3.1 years, height 172.9±8.3 cm, mass 64.74±9.81 kg) were recruited from the local community for participation in this University of Utah IRB-approved study. Each participant provided informed written consent prior to participation. Exclusion criteria were: (1) self-reported problems with balance, (2) a history of neuromotor pathology that could impair balance or locomotion, (3) any prior reconstructive surgery to the lower limbs, (4) prescribed or recreational use of psychoactive drugs for 24 h surrounding data collection, and (5) mass >136 kg (limit of shoe devices used for the perturbations).

### Procedures

**Fig. 1.**
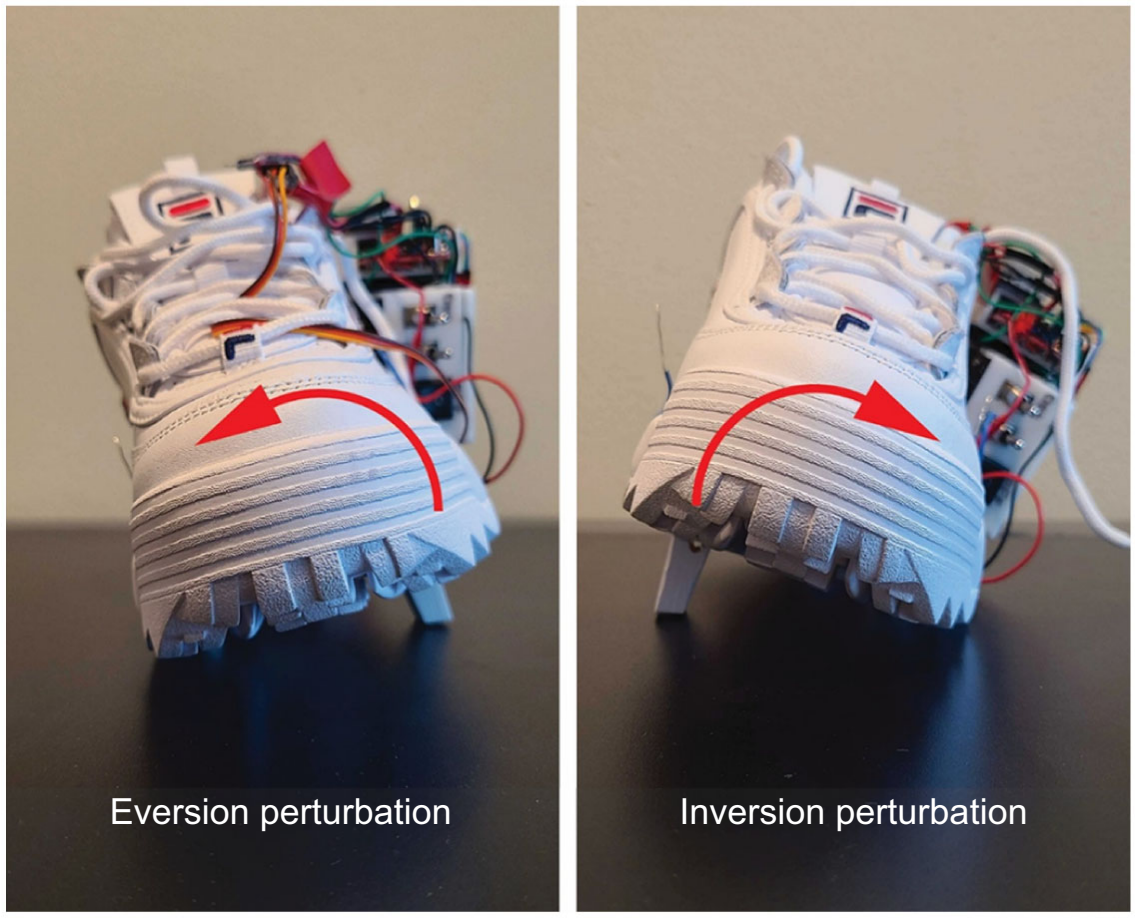
**A custom mechanized shoe with two plastic blocks recessed in the sole.** Micro-servo motors rotated these blocks out to produce either an eversion (left) or inversion (right) perturbation.

Participants were fitted with a pair of custom mechanized shoes with two plastic blocks built into the sole of the left shoe such that they would not interfere with normal gait (Fig. 1). The plastic blocks were connected to a servo motor, which would rotate one block outwards during the swing phase to elicit underfoot perturbations that either inverted or everted the foot during the next stance phase (similar to stepping on a small rock). As these perturbations are similar to unexpected terrain irregularities that individuals may experience while walking in day-to-day environments (e.g. uneven sidewalks, rocks, etc.), they offer high ecological relevance. When deployed, these plastic blocks result in approximately six degrees of inversion or eversion during straight walking (Kreter et al., 2022). The servo motors were controlled via an Arduino microprocessor and force-sensitive resistor that tracked steps. When participants were walking, the Arduino microprocessor would activate the motors randomly every 5–9 strides to deliver the underfoot perturbations. Similar designs have been used in several previous studies that have shown that these small underfoot perturbations are enough to elicit measurable changes in gait (Kim and Ashton-Miller, 2012; Kim et al., 2013; Kreter et al., 2022, 2021; Nnodim et al., 2013).

All walking trials were completed around a circular track, which had an inner radius of 1.2 m, an outer radius of 1.6 m and a thickness of 2.5 cm. To mark the progress around the track, the circle was divided into quarter-rings using colored tape. At the start of the session, participants completed a calibration trial to determine their comfortable walking speed around the track. To do so, participants were instructed to walk four laps around the track at their self-selected comfortable walking speed. The average lap time of the middle two laps determined the self-selected walking speed. The first and fourth laps were excluded to prevent influences from gait initiation or termination. Each participant was asked to maintain this comfortable pace throughout testing. Similar to prior studies, we set up a metronome to play a beat once for every quarter of their established lap time (Brown et al., 2021; Seddighi et al., 2025). Participants were instructed to complete a quarter lap every metronome beat but were not penalized for missing a mark.

**Fig. 2.**
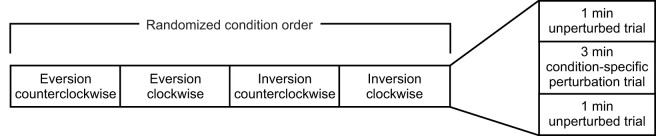
**Breakdown of the trial order for this study.** During each condition block, participants completed a 1 min unperturbed ‘acclimation’ trial, a 3 min condition-specific perturbed trial, followed by another 1 min ‘washout’ unperturbed trial.

**Fig. 3.**
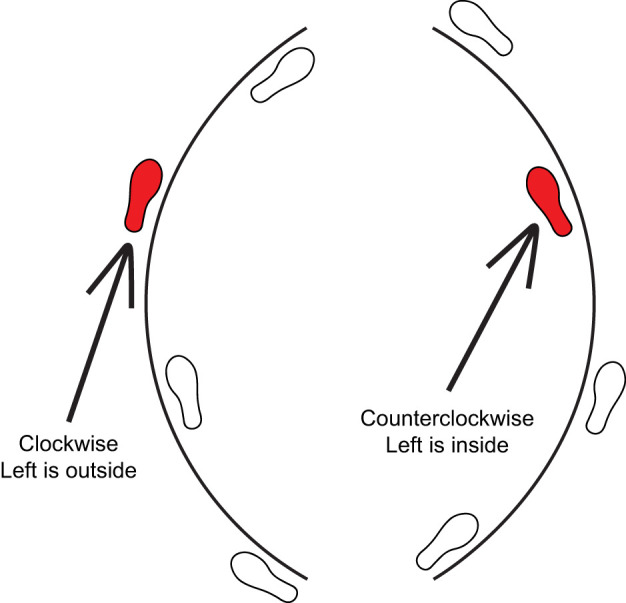
**Diagram of left steps as inside versus outside steps during turning.** We manipulated the perturbed limb by changing the direction of turning (i.e. clockwise versus counterclockwise).

Each participant completed a series of four blocks of trials where they walked either clockwise or counterclockwise and received either inversion or eversion perturbations. Each block started with a 1 min ‘acclimation’ trial with no perturbations, followed by a 3 min perturbation trial, and concluded with a 1 min ‘washout’ trial with no perturbations (Fig. 2). The order of these blocks was randomized for each participant. Because all perturbations were delivered to the left foot, only the inside foot was perturbed during counterclockwise trials and only the outside foot was perturbed during clockwise trials (Fig. 3).

A 12-camera 3D motion capture system (Nexus 2.12, Vicon, Oxford, UK; collected at 200 Hz) surrounded the walking environment and captured positional marker data from a custom marker set with 51 retroreflective markers. For our analysis, we used the bilateral retroreflective markers on the pelvis (ASIS and PSIS) and feet [second metatarsophalangeal joint (MTP), fifth MTP and calcaneus] featured in this custom marker set.

### Data analysis

Custom MATLAB codes (version R2020b, MathWorks, Natick, MA, USA) were used for data processing and data analysis. Positional data were imported and passed through a fourth-order phaseless Butterworth filter with a cutoff of 6 Hz. The body CoM position was estimated from the four pelvis markers (Havens et al., 2018) and the position and orientation of the feet were estimated from the heel, second MTP and fifth MTP markers (Havens et al., 2018). Gait events of heel strike and toe-off were calculated using the local anteroposterior (AP) maxima and minima, respectively, of the displacement of the foot relative to the pelvis (Ulrich et al., 2019). Steps were separated into inside or outside steps and were categorized as unperturbed or perturbed steps. The first four steps following a perturbation were categorized as a first, second, third or fourth recovery step (Kreter et al., 2022). Though four recovery steps were identified for each perturbation, only outcomes from the first two recovery steps were included in our results. The categorization of third and fourth recovery steps ensured that effects of the perturbation did not contaminate unperturbed steps; these steps essentially acted as ‘buffer’ steps. A rotating reference frame was aligned with the walking track to define the AP and ML directions (Ho et al., 2023). During each step, the ML direction was determined by the vector from the center of the circular track to the position of the body CoM at contralateral toe-off. The AP aspect was calculated as the cross-product of the ML aspect and a vertical vector, with the positive direction in the direction of travel (Ho et al., 2023).

To quantify the effect of the perturbations on stability, ML MoS was calculated as the distance between the extrapolated CoM (XcoM) and the lateral edge of the base of support (BoS) (Eqn 1) at the moment of contralateral toe-off (Curtze et al., 2024; Hof, 2008; Watson et al., 2021):
(1)

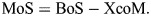
The typical MoS equation assumes simple inverted pendulum kinematics during gait where the unstable equilibrium point occurs when the body CoM is located directly over the BoS (Hof et al., 2005). However, turning at a constant radius and constant speed shifts this unstable equilibrium point away from vertical to account for centripetal force applied at the foot (Fino et al., 2016). To account for this shift, we applied a correction term based on the velocity of the CoM (*v*), gravity (*g*), the radius of the CoM (*r*_CoM_), and the height of the CoM (*L*) (Eqn 2; see Supplementary Materials and Methods for derivation):
(2)

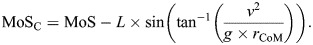
Throughout all calculations, a negative MoS or MoS_C­_ indicated the participant would fall laterally over the unstable equilibrium point, whereas a positive MoS_C_ indicated the participant would eventually fall back towards the contralateral limb.

Step width was calculated as the ML aspect of the vector connecting consecutive heel strikes, with the positive direction pointed medially from the initial heel strike in each step cycle (Ho et al., 2023). Step time was calculated as the time between consecutive heel strikes. The means of each measure (ML MoS_C_, step width and step time) for inside and outside steps were calculated across all the normal turning steps (i.e. not perturbed or recovery steps, defined as the perturbed step+4 recovery steps) within each trial. For each perturbation, we then calculated the change in ML MoS_C_, the change in step width and the change in step time relative to the mean of unperturbed steps of the same walking trial.

### Sample size justification and power analysis

We completed a power analysis based on results comparing the change in step width between unperturbed, inversion and eversion conditions using a similar mechanized shoe device (Kim and Ashton-Miller, 2012). Using an estimated effect size of 0.625 from the descriptive statistics reported by Kim and Ashton-Miller (2012), a minimum sample of 5 participants was necessary to achieve 80% power to detect differences in recovery strategies by perturbation condition.

### Statistical analysis

To test our three hypotheses, we fitted linear mixed effects models to our co-primary outcomes: the change in ML MoS_C_, the change in step width and the change in step time relative to unperturbed steps. Linear mixed effects models were fitted separately for the perturbed step, first recovery step and second recovery step. Each model included fixed effects of perturbation type (inversion versus eversion) and perturbed limb (inside versus outside). Given that the kinematic state of a given step can influence the execution of the next step (Wang and Srinivasan, 2014), the models for the recovery steps also included fixed-effects terms for the preceding step’s kinematic state to control for this step-to-step influence dependency. For the first recovery step, a single lagged term was included to represent the state of the system during the perturbed step. For the second recovery step, two lagged terms were included to represent the state of the system during the perturbed step and the first recovery step. Lagged terms were centered on 0 prior to inclusion in the model. Random intercepts for each participant accounted for repeated measures within subjects, and all models used contrast coding for fixed effects. From the linear mixed models, we estimated the mean of each variable at different levels of covariates (e.g. mean step width for inversion perturbations to the outside limb, etc.), and tested whether these mean levels were different from zero (i.e. was the perturbed/recovery step different from normal walking; H1). All analyses were completed in the Statistics and Machine Learning Toolbox in MATLAB R2020b. A significance level of 0.05 was used throughout.

## RESULTS

### Descriptive statistics

**Fig. 4.**
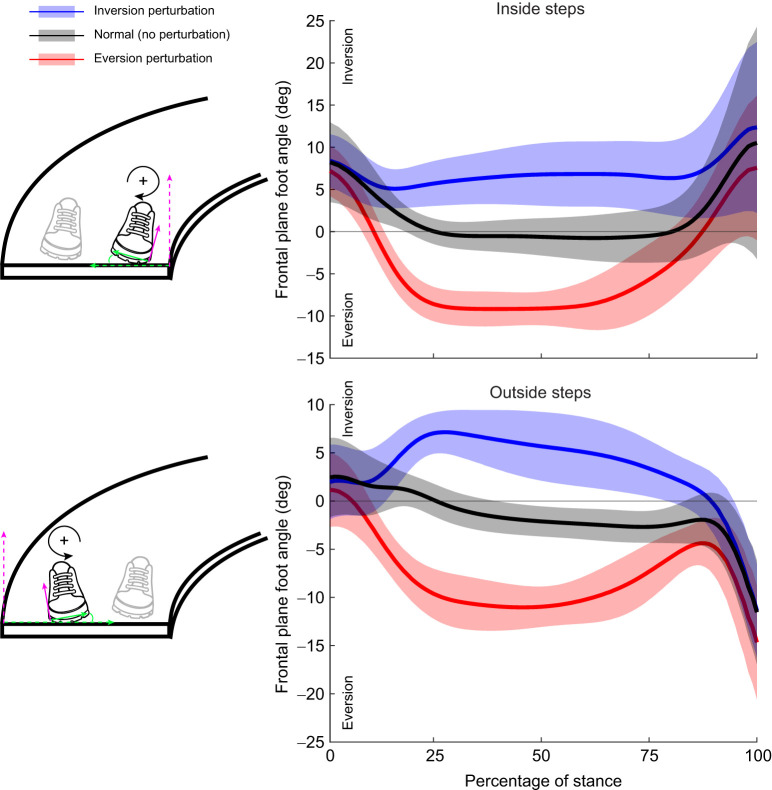
**Ensemble curves of the frontal plane angular displacement of the foot caused by perturbation for the inside (top) and outside (bottom) steps.** Angular displacements in the frontal plane are depicted relative to the local walkway reference frame (i.e. transverse plane defined by gravity and local anteroposterior direction). Solid lines indicate the mean across all normal steps (black), inversion perturbed steps (blue) and eversion perturbed steps (red) for all participants. Shaded bands depict ±1 s.d. on either side of the mean.

Participants walked at a mean±s.d. self-selected walking speed of 0.87±0.09 m s^−1^ across all trials. Within each of the four perturbation trials, participants walked at a mean±s.d. speed of 0.87±0.09 m s^−1^ (inside eversion), 0.88±0.10 m s^−1^ (inside inversion), 0.85±0.08 m s^−1^ (outside eversion) and 0.86±0.10 m s^−1^ (outside inversion). Within each trial, participants also experienced a mean±s.d. of 19.7±7.5 inside eversion perturbations, 21.9±7.5 inside inversion perturbations, 18.7±8.4 outside eversion perturbations and 20.3±7.6 outside inversion perturbations. The inversion and eversion perturbations elicited distinct changes in foot orientation throughout stance for both the inside and outside stance limb (Fig. 4). Descriptive statistics of the change in ML MoS_C_, change in step width and change in step time are presented in Table 1.

**Table 1.** Descriptive statistics of each main outcome during the perturbation trials

	Change in ML MoS_C_ (mm)	Change in step width (mm)	Change step time (ms)
Perturbed step			
Inside, eversion perturbation	−4.5±4.8	−1.4±4.4	−1.0±2.0
Inside, inversion perturbation	6.9±4.8	−0.9±13.7	−2.4±3.6
Outside, eversion perturbation	1.2±5.6	−1.1±6.8	−1.4±1.9
Outside, inversion perturbation	15.5±7.9	2.2±7.2	0.6±4.3
First recovery step			
Inside, eversion perturbation	4.4±6.3	7.6±20.2	−3.4±6.1
Inside, inversion perturbation	7.7±6.4	8.4±16.5	−8.9±3.4
Outside, eversion perturbation	−1.5±4.9	10.7±10.4	−1.0±6.8
Outside, inversion perturbation	−0.3±7.2	5.4±6.3	−7.5±3.2
Second recovery step			
Inside, eversion perturbation	−2.3±5.1	−4.9±13.3	1.2±7.6
Inside, inversion perturbation	−0.5±3.6	−3.2±10.9	0.3±9.1
Outside, eversion perturbation	2.8±4.4	14.4±13.9	−2.9±6.2
Outside, inversion perturbation	1.6±4.3	8.4±6.7	1.7±6.3

The means±s.d. of the within-participant averages of each outcome are listed for each of the perturbed step, the first recovery step and the second recovery step. ML MoS_C_, margin of stability corrected for centripetal acceleration.

### Perturbed step

**Fig. 5.**
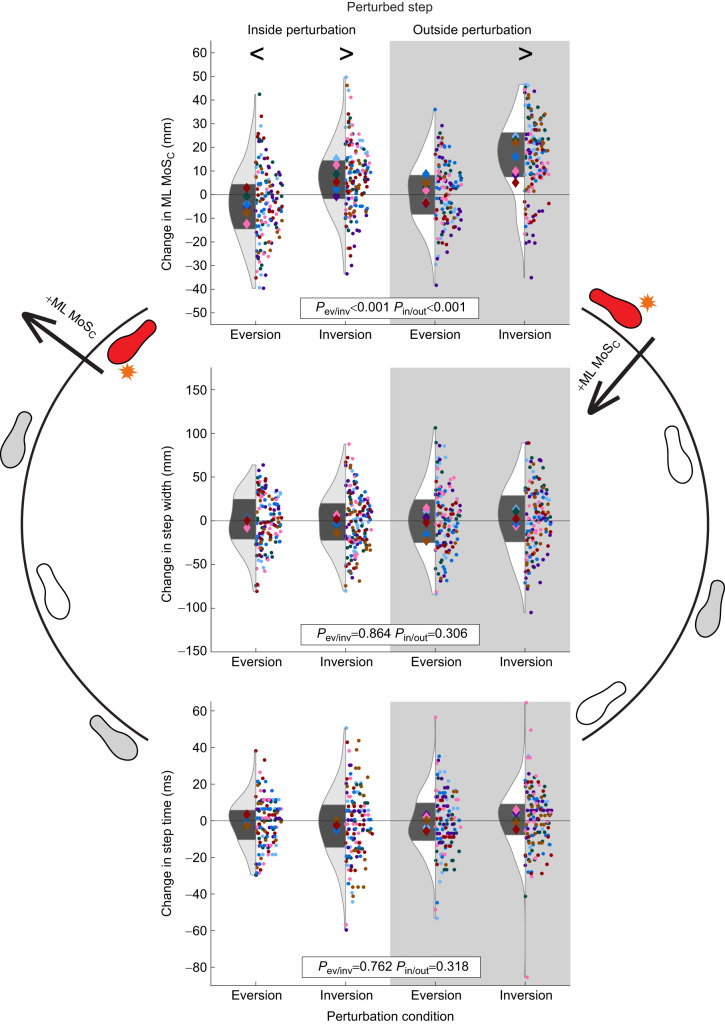
**Results of each outcome (change in ML MoS_C_, step width and step time) during each perturbed step.** These results (where ML MoS_C_ is margin of stability corrected for centripetal acceleration) were separated by eversion versus inversion perturbations, and by inside limb (white background) versus outside limb (gray background) perturbations. Each dot represents a single step and each diamond represents the mean for each participant. The diamonds and dots are color coded per participant. In the violin plots, the dark gray area represents the interquartile range of all participants (*n*=7). *P*-values are provided for the effects of perturbation type (*P*_ev/inv_) and perturbed limb (*P*_in/out_). Symbols > and < indicate whether individual levels were significantly greater than or less than 0, respectively, based on the adjusted mean levels of each variable from the linear mixed model.

Compared with that for the unperturbed gait, the ML MoS_C_ during the perturbed step decreased during inside eversion perturbations (*P*<0.001; Tables 1 and 2, Fig. 5) but increased during inside inversion perturbations (*P*<0.001). While ML MoS_C_ did not differ from that for the unperturbed gait during outside eversion perturbations (*P*=0.103), ML MoS_C_ increased during outside inversion perturbations (*P*<0.001). Step width and step time of the perturbed step were not different between the perturbed step and unperturbed gait for any condition (step width *P*=0.860, step time *P*=0.182).

**Table 2.** Coefficients and results from the linear mixed models for the change from the mean ML MoS_C_, step width and step time during each perturbed step and the two subsequent recovery steps

	Change in ML MoS_C_ (mm)		Change in step width (mm)		Change step time (ms)	
	β (95% CI)	*P*	β (95% CI)	*P*	β (95% CI)	*P*
Perturbed step (d.f.=561)						
Intercept (mean effect of perturbation)	4.8 (3.5, 6.0)	**<0.001**	0.3 (−2.6, 3.2)	0.860	−1.0 (−2.3, 0.4)	0.182
Perturbation limb (inside versus outside)	−3.5 (−4.7, −2.3)	**<0.001**	−1.5 (−4.4, 1.4)	0.306	−0.7 (−2.1, 0.7)	0.318
Perturbation type (eversion versus inversion)	−6.4 (−7.7, −5.2)	**<0.001**	−0.3 (−3.2, 2.6)	0.864	−0.2 (−1.6, 1.2)	0.762
First recovery step (d.f.=560)						
Intercept (mean effect of perturbation)	2.7 (1.5, 3.9)	**<0.001**	8.3 (4.9, 11.6)	**<0.001**	−5.4 (−6.8, −4.0)	**<0.001**
Perturbation limb (inside versus outside)	3.6 (2.4, 4.9)	**<0.001**	0.7 (−2.6, 4.1)	0.666	−1.2 (−2.5, 0.2)	0.095
Perturbation type (eversion versus inversion)	−1.1 (−2.3, 0.14)	0.083	1.1 (−2.3, 4.4)	0.531	2.7 (1.3, 4.0)	**<0.001**
*n*−1 lag-term effect (perturbed step)	0.2 (0.1, 0.3)	**<0.001**	0.1 (0.0, 0.1)	0.302	0.5 (0.4, 0.6)	**<0.001**
Second recovery step (d.f.=559)						
Intercept (mean effect of perturbation)	0.6 (−0.5, 1.6)	0.272	3.5 (0.6, 6.5)	**0.019**	0.5 (−3.9, 4.9)	0.830
Perturbation limb (inside versus outside)	−1.8 (−2.9, −0.8)	**<0.001**	−7.2 (−10.2, −4.3)	**<0.001**	0.2 (−0.9, 1.3)	0.732
Perturbation type (eversion versus inversion)	−0.0 (−1.0, 1.1)	0.956	1.4 (−1.5, 4.4)	0.340	−0.5 (−1.7, 0.6)	0.388
*n*−2 lag-term effect (perturbed step)	−0.2 (−0.2, −0.1)	**<0.001**	−0.1 (−0.2, −0.5)	**0.003**	0.2 (0.1, 0.3)	**<0.001**
*n*−1 lag-term effect (first recovery step)	0.1 (0.1, 0.2)	**<0.001**	−0.0 (−0.1, 0.0)	0.378	0.2 (0.2, 0.3)	**<0.001**

Fixed effects were perturbed limb (inside versus outside) and perturbation type (eversion versus inversion). The models for the first recovery step included a fixed-effect of the *n*−1 (perturbed) step. The models for the first recovery step included fixed-effects for both the *n*−2 (perturbed) and *n*−1 (first recovery) steps. Bold *P*-values indicate statistical significance (α=0.05). All β coefficients were contrast coded with eversion and inside limbs effects coded with a +1, and inversion and outside limb effects coded with a −1. CI, confidence interval.

The effect of unexpected underfoot perturbations on ML MoS_C_ during the perturbed step varied depending on the type of perturbation and the stance limb (Tables 1 and 2, Fig. 5). Specifically, outside limb perturbations increased ML MoS_C_ more than inside limb perturbations (β=−3.5 mm, *P*<0.001; Table 2, Fig. 5). Similarly, inversion perturbations produced a more positive change in ML MoS_C_ compared with eversion perturbations (β=−6.4 mm, *P*<0.001). Perturbations did not affect the step width or step time during the perturbed step.

### First recovery step

**Fig. 6.**
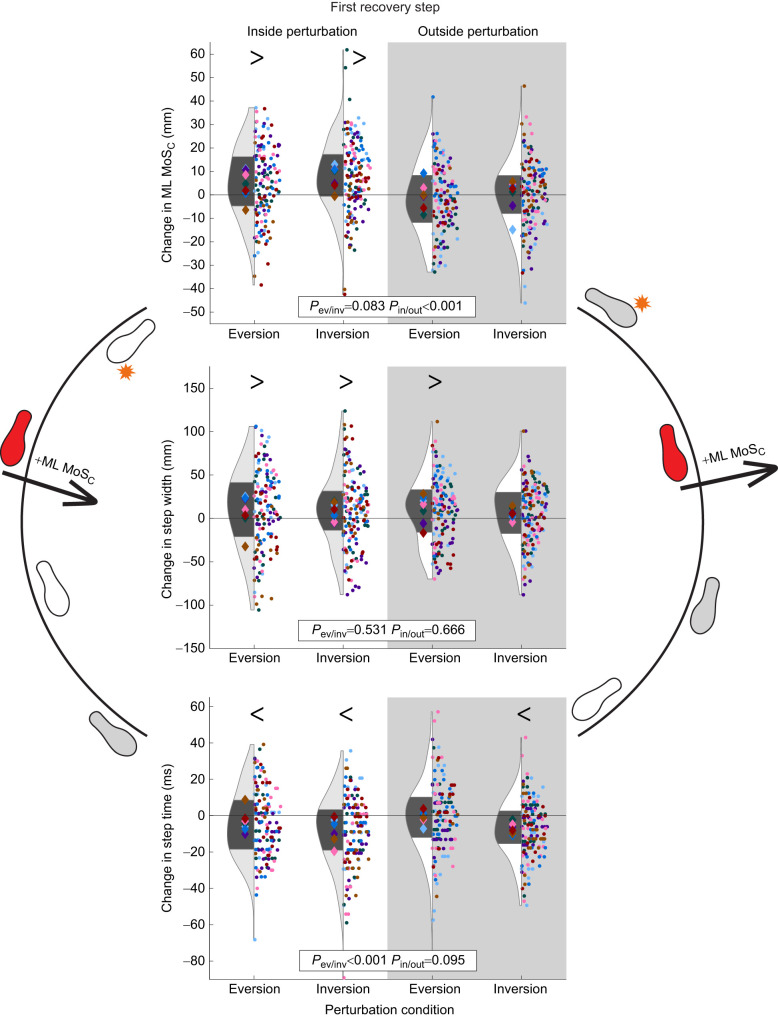
**Results of each outcome (change in ML MoS_C_, step width and step time) during the first recovery step after each perturbation.** These results were separated by eversion versus inversion perturbations, and by inside limb (white background) versus outside limb (gray background) perturbations. Each dot represents a single step and each diamond represents the mean for each participant. The diamonds and dots are color coded per participant. In the violin plots, the dark gray area represents the interquartile range of all participants (*n*=7). *P*-values are provided for the effects of perturbation type (*P*_ev/inv_) and perturbed limb (*P*_in/out_). Symbols > and < indicate whether individual levels were significantly greater than or less than 0, respectively, based on the adjusted mean levels of each variable from the linear mixed model.

ML MoS_C_ increased relative to that for the unperturbed gait after all inside limb perturbations (inside eversion *P*<0.001; inside inversion *P*<0.001) but was not different from that for the unperturbed gait following outside limb perturbations (outside eversion *P*=0.072; outside inversion *P*=0.872). Participants took a wider first recovery step after all perturbations (inside eversion *P*=0.001, inside inversion *P*=0.006, outside eversion *P*=0.005, outside inversion *P*=0.029; Tables 1 and 2, Fig. 6). Participants also took a faster first recovery step after inside eversion (*P*=0.002), inside inversion (*P*<0.001) and outside inversion perturbations (*P*<0.001), but not after outside eversion perturbations (*P*=0.210).

Perturbation to the inside limb increased the ML MoS_C_ during the first recovery step compared with outside limb perturbation (β=3.6 mm, *P*<0.001). The change in step time during the first recovery step differed between perturbation types, with faster step times after inversion perturbations compared with eversion perturbations (β=2.7 ms, *P*<0.001; Table 2, Fig. 6).

While there were significant *n*−1 lag-term effects on ML MoS_C_ (*P*<0.001) and step time (*P*<0.001), these effects did not change any of the effects by perturbation type and perturbed limb (see Table S1).

### Second recovery step

**Fig. 7.**
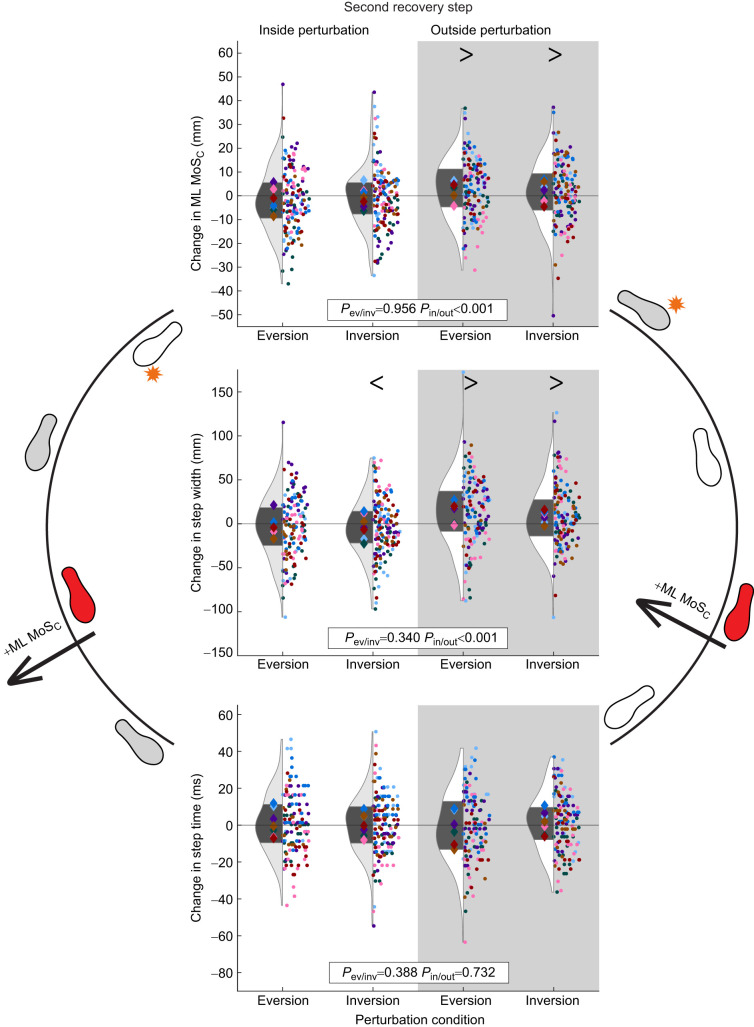
**Results of each outcome (change in ML MoS_C_, step width and step time) during the second recovery step after each perturbation.** These results were separated by eversion versus inversion perturbations, and by inside limb (white background) versus outside limb (gray background) perturbations. Each dot represents a single step and each diamond represents the mean for each participant. The diamonds and dots are color coded per participant. In the violin plots, the dark gray area represents the interquartile range of all participants (*n*=7). *P*-values are provided for the effects of perturbation type (*P*_ev/inv_) and perturbed limb (*P*_in/out_). Symbols > and < indicate whether individual levels were significantly greater than or less than 0, respectively, based on the adjusted mean levels of each variable from the linear mixed model.

ML MoS_C_ during the second recovery step was greater than that for unperturbed walking after all outside perturbations (outside eversion *P*=0.010, outside inversion *P*=0.010), but not different from normal walking during the second recovery step after inside perturbations (*P*=0.159). Participants also took a wider second recovery step following outside eversion (*P*<0.001) and outside inversion perturbations (*P*<0.001), and a narrower second recovery step after inside inversion perturbations compared with unperturbed walking (*P*=0.043; Tables 1 and 2, Fig. 7). Step time for the second recovery step was not different from that for normal walking for any condition or direction (*P*=0.617).

Perturbations to the outside limb increased ML MoS_C_ during the second recovery step compared with perturbations to the inside limb (β=−1.8 mm, *P*=0.002). The change in step width during the second recovery step also differed by perturbed limb, with perturbations to the outside limb leading to wider second recovery steps compared with perturbations to the inside limb (β=−7.2 mm, *P*<0.001; Table 2, Fig. 7). Perturbation type (inversion/eversion) had no effect on ML MoS_C_ (*P*=0.956), step width (*P*=0.340) or step time (*P*=0.388) of the second recovery step.

While there were significant *n*−2 lag-term effects on ML MoS_C_ (*P*<0.001), step width (*P*<0.001) and step time (*P*<0.001), as well as *n*−1 lag-term effects on ML MoS_C_ (*P*<0.001) and step time (*P*<0.001), these effects did not change any of the effects by perturbation type and perturbed limb (see Table S1).

## DISCUSSION

This study aimed to investigate the effect of unexpected underfoot perturbations on stability during turning. Our three hypotheses were that: (1) participants' ML MoS_C_ would deviate from normal walking during perturbed steps and up to two recovery steps (H1), (2) participants would make opposite changes in their step width and step time in response to inversion and eversion perturbations (H2), and (3) participants would make larger changes in their step width and step time in response to outside limb perturbations compared with inside limb perturbations (H3). We found that ML MoS_C_ significantly changed during all perturbed steps except eversion perturbations to the outside limb. ML MoS_C_ was greater, compared with that for unperturbed turning gait, during the first recovery step after inside limb perturbations and during the second recovery step after an outside eversion perturbation. We also found that outside limb perturbations elicited more changes in step width and step time than inside limb perturbations. The only consistent difference between perturbation types (i.e. inversion versus eversion) across limbs was observed in the step time of the first recovery step; participants took a faster step after an inversion perturbation than after an eversion perturbation, regardless of the perturbed limb. These results suggest that perturbations during turning create different effects based on the type of perturbation and the stance limb that is perturbed, and that contributions to stability recovery during turning may be asymmetrical.

Changes in ML MoS_C_ suggest perturbations during turning can differentially affect stability and forward progression along a desired path. ML MoS_C_ decreased during inside eversion perturbations but increased during inversion perturbations. However, such increases do not necessarily mean participants were less likely to fall during an inversion underfoot perturbation. A more positive ML MoS_C_ here indicates greater medial instability (Curtze et al., 2024), which also necessitates recovery strategies to re-establish normal walking. Importantly, the effect of greater medial instability during turning differed by inside and outside limb (see Figs 5–7). During single-limb stance with a fixed base of support, greater medial instability for the outside limb indicates increased instability towards the center of the turn, while greater medial instability for the inside limb indicates increased instability away from the center of the turn. With this perspective, the change in ML MoS_C_ during both inside eversion perturbations and outside inversion perturbations indicates greater relative instability towards the center of the circle. In contrast, the change in ML MoS_C_ during inside inversion perturbations indicates greater relative instability away from the center of the circle. Therefore, inversion perturbations consistently elicit changes in ML MoS_C_ towards the contralateral limb, but these changes have different effects on CoM progression around a curve based on whether the inside or outside limb is perturbed.

Regardless of the limb laterality or perturbation type, participants did not return to normal gait until they could make a corrective step with their outside limb, suggesting the recovery limb (inside/outside) plays a significant role. Participants took wider and faster first recovery steps after perturbations to the inside limb (i.e. where the first recovery step was with the outside limb). These corrections were sufficient to return to normal gait at the second recovery step. In contrast, participants took two steps to recover from perturbations to the outside limb, but the specific recovery strategy depended on perturbation type. That is, during the first recovery step following a perturbation to the outside limb (i.e. where the first recovery step was with the inside limb), participants exhibited wider recovery steps for outside eversion perturbations, and both wider and faster recovery steps for outside inversion perturbations. Further, participants continued to exhibit a wider second step after perturbations to the outside limb (i.e. where the second recovery step was with the outside limb).

**Fig. 8.**
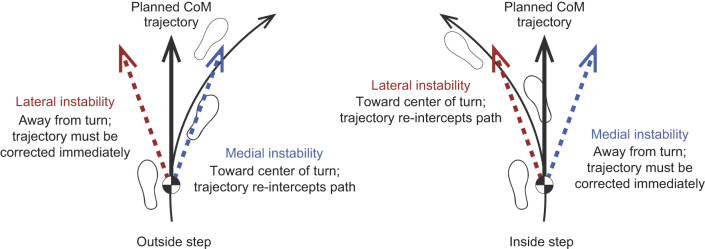
**Illustration describing the change in center of mass (CoM) trajectory from lateral instability (red) versus medial instability (blue) on outside steps (left) compared with inside steps (right).** Lateral instability during an outside step and medial instability during an inside step both indicate an altered trajectory that deviates from the planned trajectory and must be immediately corrected. However, medial instability during an outside step and lateral instability during an inside step both indicate an altered trajectory that re-intercepts the planned trajectory at a later point. These altered trajectories may not require immediate correction.

The different recovery strategies between perturbations to inside and outside limbs may also be explained as an attempt to limit the curvature of the CoM trajectory. Assuming that the optimal instantaneous CoM trajectory is tangent to the curvature of the path, a perturbation that produces instability away from the center of the circle creates a new CoM trajectory that will never intercept the circle unless corrected (Fig. 8). Immediately correcting this perturbation may be most effective to limit the CoM's divergence away from the circular path. Alternatively, perturbations that generate greater instability toward the center of the circle create a new CoM trajectory that will eventually re-intercept the circle (Fig. 8). So long as the perturbation does not pose an immediate threat of falling, a person may choose to correct the CoM trajectory on either the first or the second recovery step. Here, participants made adjustments to step width throughout the first outside recovery step (i.e. one step after perturbations to the inside limb, or two recovery steps after perturbations to the outside limb). Overall, these results suggest that adjustments to outside steps may be preferred over adjustments to inside steps when recovering from perturbations during turning (Rasmussen et al., 2022).

Alternatively, limitations of the circular walkway may explain this delay in recovery after an outside limb perturbation. We found that participants tended to walk near the inside border of the walkway and, as such, they may not have had enough space to make the step width adjustment that they normally would have in an uncontrolled environment. This interpretation seems unlikely, given that similar results report bias towards recovering on the outside limb during unconstrained turning (Kreter and Fino, 2024; Rasmussen et al., 2022) and that humans tend to be biased against trajectories that force sharper turns based on energetic demands (Brown et al., 2021).

Notably, the changes in ML MoS_C_, step width and step time elicited by the perturbation were small (i.e. on the scale of millimeters and milliseconds). Despite their small magnitude, these changes in ML MoS_C_ and step width are of a comparable magnitude to those seen in previous studies that used similar devices to deliver underfoot perturbations (Kim and Ashton-Miller, 2012; Kreter et al., 2022), and that have shown clinical significance in predicting falls (Allet et al., 2014). We suspect such differences would extrapolate to larger magnitude perturbations that require larger changes in gait, but future research should consider different types (e.g. at different locations along the foot, or different mechanisms) and magnitudes of perturbations.

The primary limitation of this study is the small sample size. Despite our power analysis, the small sample size herein may not be generalizable to different populations. Future studies should seek to confirm and extend these findings in a larger, more representative population. Another limitation of this study is that all of the perturbations were delivered to each participant's left foot, regardless of the trial condition. We deliberately made this decision because of the technical challenges associated with syncing pseudo-random perturbation devices on each foot. By isolating the perturbation to one limb, we ensured that perturbed steps and recovery steps never overlapped and could be assessed independently. To avoid overburdening our participants, we chose not to repeat each trial condition for each foot and instead maintained left-foot-only perturbations across all of our data collections. However, it is possible the differences in limb laterality could influence the results observed here. The outcomes measured in this study are also all discrete spatiotemporal gait measures, which cannot describe the continuous, multi-joint neuromechanical control strategies that contribute to reactive balance. Future work should investigate how the nervous system coordinates recovery from a perturbation during turning gait using continuous neuromechanical measures of control, including measures of muscle activity and joint torques.

Additionally, while the kinematic-based algorithms for detecting heel strike and toe-off events used here are well validated (Ulrich et al., 2019), they are relatively less precise than methods that rely on ground reaction forces. However, their use in this study was a necessity because of the turning component of the experimental design, which made the use of an instrumented treadmill impossible, and the use of in-ground force plates highly inefficient (i.e. force plates would need to align with the turning trajectory, participants would need to step accurately on them for three consecutive steps, and perturbations would always need to occur at or around the first force plate in the series, increasing the perturbation's predictability). In contrast, the use of the kinematic-based event detection allowed for every step to be included in the analysis, maintained the perturbation's unpredictable nature and limited the overall burden on participants volunteering in the study.

Finally, turns in everyday life are often discrete events and not continuous, as they were in this experiment. The use of a continuous turn around a circle allowed us to capture many repeated perturbations per trial in a setting that resembled steady-state turning, much like past work using treadmills has examined perturbations during steady-state walking. But, this choice eliminated unique features of turning, such as anticipatory postural adjustments and segmental reorientation patterns that are evident when navigating one's workplace or home (Xu et al., 2004; Bernardin et al., 2012). Future work should investigate how perturbations during the approach from straight-line walking into a turn, the midpoint of a turn or the transition out of a turn back into straight-line walking affect the control of upright stability.

### Conclusion

Underfoot perturbations disrupted ML MoS_C_ differently across perturbation type, and the change in ML MoS_C_ was dependent on the limb being perturbed. After these underfoot perturbations during turning, participants employed similar recovery strategies to those previously seen in linear walking (i.e. increased step width and decreased step time) (Kim et al., 2013; Kreter et al., 2022). However, the magnitude of these changes in foot placement and timing was also largely determined by the perturbed limb, as participants tended to wait until an outside limb recovery step to correct the ML MoS_C_. These results demonstrate that outside steps may be more important for maintaining and recovering stability during turning compared with inside steps, which may be used more for maintenance of the status quo rather than corrective control.

## Supplementary Material

10.1242/jexbio.251471_sup1 Supplementary information
